# Supplementary material for: Pyruvate Dehydrogenase Kinase 1 inhibition mediated oxidative phosphorylation enhancement in cartilage promotes osteoarthritis progression
Source: BMC Musculoskelet Disord. 2023 Jul 20;24:597. doi: 10.1186/s12891-023-06585-6 (PMC10357736; doi:10.1186/s12891-023-06585-6)
Supplement: Supplementary file 1 — Additional file 1. [file 12891_2023_6585_MOESM1_ESM.zip › KEGG.Gsea.1653622432464/KEGG_PROPANOATE_METABOLISM.html]

Details for gene set KEGG\_PROPANOATE\_METABOLISM[GSEA]

|  || Dataset | 55457\_collapsed\_to\_symbols.cls.cls #OA\_versus\_normal.cls.cls #OA\_versus\_normal\_repos |
| Phenotype | cls.cls#OA\_versus\_normal\_repos |
| Upregulated in class | OA |
| GeneSet | KEGG\_PROPANOATE\_METABOLISM |
| Enrichment Score (ES) | 0.43532774 |
| Normalized Enrichment Score (NES) | 1.5457151 |
| Nominal p-value | 0.028423773 |
| FDR q-value | 0.05103425 |
| FWER p-Value | 0.697 |
Table: GSEA Results Summary

  

Fig 1: Enrichment plot: KEGG\_PROPANOATE\_METABOLISM      
 Profile of the Running ES Score & Positions of GeneSet Members on the Rank Ordered List

  

| SYMBOL | TITLE | RANK IN GENE LIST | RANK METRIC SCORE | RUNNING ES | CORE ENRICHMENT || 1 | PCCB | propionyl-CoA carboxylase subunit beta [Source:HGNC Symbol;Acc:HGNC:8654] | 29 | 1.057 | 0.1401 | Yes |
| 2 | ALDH2 | aldehyde dehydrogenase 2 family member [Source:HGNC Symbol;Acc:HGNC:404] | 1003 | 0.517 | 0.1341 | Yes |
| 3 | ACAT1 | acetyl-CoA acetyltransferase 1 [Source:HGNC Symbol;Acc:HGNC:93] | 1017 | 0.513 | 0.2022 | Yes |
| 4 | ALDH3A2 | aldehyde dehydrogenase 3 family member A2 [Source:HGNC Symbol;Acc:HGNC:403] | 1263 | 0.465 | 0.2458 | Yes |
| 5 | SUCLG2 | succinate-CoA ligase GDP-forming subunit beta [Source:HGNC Symbol;Acc:HGNC:11450] | 1472 | 0.432 | 0.2879 | Yes |
| 6 | HIBCH | 3-hydroxyisobutyryl-CoA hydrolase [Source:HGNC Symbol;Acc:HGNC:4908] | 1526 | 0.424 | 0.3409 | Yes |
| 7 | ECHS1 | "enoyl-CoA hydratase, short chain 1 [Source:HGNC Symbol;Acc:HGNC:3151]" | 1925 | 0.369 | 0.3598 | Yes |
| 8 | ALDH7A1 | aldehyde dehydrogenase 7 family member A1 [Source:HGNC Symbol;Acc:HGNC:877] | 2158 | 0.343 | 0.3880 | Yes |
| 9 | SUCLG1 | succinate-CoA ligase GDP/ADP-forming subunit alpha [Source:HGNC Symbol;Acc:HGNC:11449] | 2269 | 0.330 | 0.4239 | Yes |
| 10 | ALDH9A1 | aldehyde dehydrogenase 9 family member A1 [Source:HGNC Symbol;Acc:HGNC:412] | 2795 | 0.270 | 0.4195 | Yes |
| 11 | SUCLA2 | succinate-CoA ligase ADP-forming subunit beta [Source:HGNC Symbol;Acc:HGNC:11448] | 3228 | 0.228 | 0.4166 | Yes |
| 12 | MMUT | methylmalonyl-CoA mutase [Source:HGNC Symbol;Acc:HGNC:7526] | 3362 | 0.215 | 0.4353 | Yes |
| 13 | HADHA | hydroxyacyl-CoA dehydrogenase trifunctional multienzyme complex subunit alpha [Source:HGNC Symbol;Acc:HGNC:4801] | 4163 | 0.141 | 0.3922 | No |
| 14 | MLYCD | malonyl-CoA decarboxylase [Source:HGNC Symbol;Acc:HGNC:7150] | 4852 | 0.083 | 0.3500 | No |
| 15 | ACADM | acyl-CoA dehydrogenase medium chain [Source:HGNC Symbol;Acc:HGNC:89] | 4983 | 0.074 | 0.3499 | No |
| 16 | LDHAL6B | lactate dehydrogenase A like 6B [Source:HGNC Symbol;Acc:HGNC:21481] | 5040 | 0.070 | 0.3549 | No |
| 17 | ALDH6A1 | aldehyde dehydrogenase 6 family member A1 [Source:HGNC Symbol;Acc:HGNC:7179] | 5560 | 0.028 | 0.3183 | No |
| 18 | ACAT2 | acetyl-CoA acetyltransferase 2 [Source:HGNC Symbol;Acc:HGNC:94] | 6291 | -0.027 | 0.2652 | No |
| 19 | ALDH1B1 | aldehyde dehydrogenase 1 family member B1 [Source:HGNC Symbol;Acc:HGNC:407] | 6551 | -0.046 | 0.2512 | No |
| 20 | PCCA | propionyl-CoA carboxylase subunit alpha [Source:HGNC Symbol;Acc:HGNC:8653] | 6627 | -0.052 | 0.2524 | No |
| 21 | ABAT | 4-aminobutyrate aminotransferase [Source:HGNC Symbol;Acc:HGNC:23] | 6804 | -0.063 | 0.2472 | No |
| 22 | LDHC | lactate dehydrogenase C [Source:HGNC Symbol;Acc:HGNC:6544] | 7603 | -0.127 | 0.2024 | No |
| 23 | LDHB | lactate dehydrogenase B [Source:HGNC Symbol;Acc:HGNC:6541] | 7718 | -0.135 | 0.2117 | No |
| 24 | ACSS3 | acyl-CoA synthetase short chain family member 3 [Source:HGNC Symbol;Acc:HGNC:24723] | 7821 | -0.143 | 0.2230 | No |
| 25 | EHHADH | enoyl-CoA hydratase and 3-hydroxyacyl CoA dehydrogenase [Source:HGNC Symbol;Acc:HGNC:3247] | 7968 | -0.153 | 0.2322 | No |
| 26 | ACACA | acetyl-CoA carboxylase alpha [Source:HGNC Symbol;Acc:HGNC:84] | 9177 | -0.246 | 0.1715 | No |
| 27 | ACACB | acetyl-CoA carboxylase beta [Source:HGNC Symbol;Acc:HGNC:85] | 10955 | -0.425 | 0.0907 | No |
| 28 | LDHA | lactate dehydrogenase A [Source:HGNC Symbol;Acc:HGNC:6535] | 11134 | -0.448 | 0.1372 | No |
Table: GSEA details [plain text format]

  

Fig 2: KEGG\_PROPANOATE\_METABOLISM      
 Blue-Pink O' Gram in the Space of the Analyzed GeneSet

  

Fig 3: KEGG\_PROPANOATE\_METABOLISM: Random ES distribution      
 Gene set null distribution of ES for **KEGG\_PROPANOATE\_METABOLISM**
